# Recent Developments in Polymer Nanocomposites for Bone Regeneration

**DOI:** 10.3390/ijms24043312

**Published:** 2023-02-07

**Authors:** Mohamed Abbas, Mohammed S. Alqahtani, Roaa Alhifzi

**Affiliations:** 1 Electrical Engineering Department, College of Engineering, King Khalid University, Abha 61421, Saudi Arabia; 2 Research Center for Advanced Materials Sciences (RCAMS), King Khalid University, Abha 61413, Saudi Arabia; 3 BioImaging Unit, Space Research Centre, University of Leicester, Michael Atiyah Building, Leicester LE1 7RH, UK; 4 Radiological Sciences Department, College of Applied Medical Sciences, King Khalid University, Abha 61421, Saudi Arabia

**Keywords:** bone regeneration, nanocomposites, biomaterials, scaffolds, ceramic matrix composites

## Abstract

Most people who suffer acute injuries in accidents have fractured bones. Many of the basic processes that take place during embryonic skeletal development are replicated throughout the regeneration process that occurs during this time. Bruises and bone fractures, for example, serve as excellent examples. It almost always results in a successful recovery and restoration of the structural integrity and strength of the broken bone. After a fracture, the body begins to regenerate bone. Bone formation is a complex physiological process that requires meticulous planning and execution. A normal healing procedure for a fracture might reveal how the bone is constantly rebuilding as an adult. Bone regeneration is becoming more dependent on polymer nanocomposites, which are composites made up of a polymer matrix and a nanomaterial. This study will review polymer nanocomposites that are employed in bone regeneration to stimulate bone regeneration. As a result, we will introduce the role of bone regeneration nanocomposite scaffolds, and the nanocomposite ceramics and biomaterials that play a role in bone regeneration. Aside from that, recent advances in polymer nanocomposites might be used in a variety of industrial processes to help people with bone defects overcome their challenges will be discussed.

## 1. Introduction

Fractures mend over time, resulting in the formation of bone. Several physiological systems regulate bone development. Falls, traffic accidents, and contact sports cause most fractures, which take a long time to heal. However, certain medical disorders or prolonged stress may increase the risk of a certain fracture. Fractures are characterized by their appearance, cause, and body component. These fissures are tectonic. Mineral dissolution causes “dissolving fractures” or tectonic fractures. Tension, shear, and even microscopic fissures can be tectonic. Dissolution fractures have two subtypes. Weathering or internal collapse causes these fissures [1]. Membranes exist in a wide range of shapes and sizes, which affects both their mechanical and therapeutic properties. Because of their biocompatibility and wound-healing properties, collagen membranes are employed in medical and dental applications. Collagen membranes have also been employed to help direct the process of bone regeneration [2]. In the case of transplant scarcity, it is advised that bone tissue be rebuilt. Bioactive and osteoinductive scaffolds are good for bone regeneration [3]. Zinc is also beneficial for bone development and repair.

**Figure 1:**
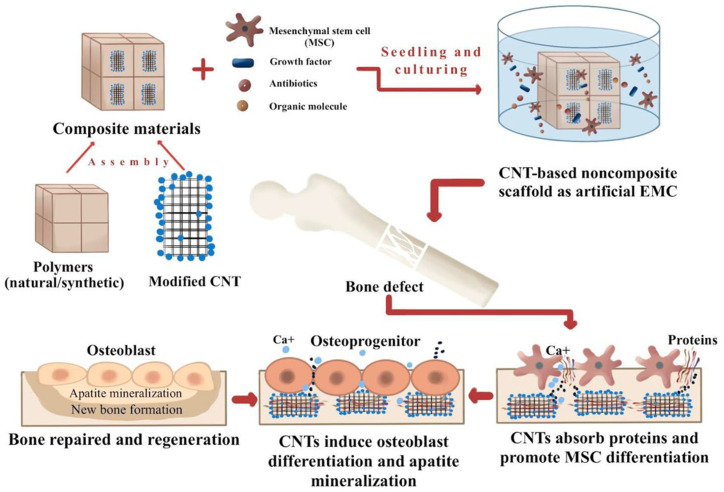
The process of bone regeneration using MSCs.

On the other hand, the mechanism by which zinc enhances bone development, homeostasis, and regeneration remains unclear [4]. To repair and replace bone, the host’s mesenchymal stem cells (MSCs) must be able to distinguish between various forms of bone. In critical-sized wounds, the growth factor BMP2 (bone morphogenetic protein 2) is used to enhance wound healing because of the risk of an ectopic pregnancy and dose problems [5]. A new bone healing approach has been developed using MSC secretome functional extracellular vesicles (EVs). For the recurrence of malignant bone tumors and bone loss following surgery, multimodal treatment platforms are critical. The MSC-assisted bone healing process is shown in Figure 1. The combining of natural polymers with carbon nanotubes has been shown in Figure 1 to create a nanocomposite material, as seen in this picture. This material might be utilized to make a carbon nanotube-based nanocomposite scaffold with mesenchymal stem cells. In this milieu, carbon nanotubes absorb proteins and drive MSC differentiation. After that, the bone would be repaired and reconstructed [6].

For effective bone regeneration treatment, drug delivery systems and cell differentiation carriers are necessary. It is normal practice to use the patient’s own tissue, which is referred to as an “autograft”, when reconstructive surgery is performed on a patient. This procedure is known as “autografting.” It takes longer for allograft tissue, which is tissue that has been removed from one person and transplanted into another, to become fully integrated into the body of the person who has received it. Meanwhile, a xenograft refers to the transplantation of an organ or tissue from a donor of a species that is not the same as the recipient. In recent years, biodegradable polymers have gained favor because of their quick, localized absorption and ability to substitute autologous bone [7]. Hydroxyapatite and tricalcium phosphate are used to rebuild bone in therapeutic settings. Tissue engineering and other technical advances are helpful in satisfying the high demand for bone autograft replacements in clinical practice. Biodegradable matrices are required to protect cells and promote tissue growth until transplantation. Biocompatibility and bioactivity often outperform polymers. As complicated supporting materials, hydrogel-based systems and fibrous or porous scaffolds [8] have been discovered and developed. A biopolymer matrix encases bioactive and biodegradable nanoparticles. It has been shown that a polymeric nanocomposite biomaterial may help with bone tissue repair [9].

Tricalcium phosphate, hyaluronic acid, and nanosilver from corn silk extract are included in the hydrogels (CSE-Ag NPs). Microwaves were used to biosynthesize silver spherical nanoparticles in an organic solvent-free solution. Thermosensitive hydrogels gel at temperatures close to human body temperatures [10]. Encapsulated pharmaceuticals or growth factors have been used to improve bone repair [11]. Bone loss may be caused for a multitude of reasons. The extracellular matrix has an impact on mature bone function, and the surrounding environment includes a variety of mechanical and biological elements (ECM). The ECM in the bone may activate osteoclasts, causing them to absorb new bone. The osteoinductive, osteoconductive, and osteogenic properties of ECM-based scaffolds have recently been investigated. The addition of an ECM to the implantation site aids tissue growth [12].

Bone development is induced by a biodegradable and photocrosslinkable hydrogel. Bone healing in craniofacial fractures may be difficult due to the wet environment in the mouth [13]. miRNAs generated by polarized macrophage extracellular vesicles (EVs) are required for paracrine bone repair [14]. The immune system is complex, with several immune cells releasing both pro- and anti-inflammatory cytokines. Prior to the regenerative or degenerative stages of bone fracture repair, immune cell–cell interactions generate acute inflammation. Macrophages are vital for wound healing and immune system function [15]. According to some research [16], controlled release of the core-shell nanofiber structure might aid in promoting bone healing. The immune response to bone biomaterials has an impact on the destiny of the scaffold and the result of the regeneration process. Inhibition of osteogenic differentiation and local immunological response by nanoscale biomaterial interfaces has been shown [17].

Magnesium ions (Mg^2+^) are bioactive and have been shown to aid in the regeneration of bone tissue. When Mg^2+^ is released under strict control in bone tissue engineering, it has the potential to have a major impact on bone regeneration. Biodegradable microspheres are being used to manage the release of bioactive chemicals [18]. Several bone regeneration products, including bone grafting, barrier membranes, bioactive chemicals, and cell treatments, have shown an additional benefit [19]. Osteoinductive or osteogenic components may be placed into them and released in a variety of ways to stimulate osteogenesis [20]. The purpose of this paper is to present and examine in depth, the current trend of polymer nanocomposites used in the bone regeneration process, which is now in its early stages. In this section, three forms of composites will be discussed: nanocomposite scaffolds, nanocomposite ceramics, and several types of biomaterials. Nanocomposite scaffolds are scaffolds made of nanoscale particles that are bonded together.

## 2. Regeneration of Bones Using Nanocomposite Scaffolds

A nanocomposite combining chitosan (CS) and polyhedral oligomeric silsesquioxanes (OctaTMA-POSS) nanoparticles has been developed for bone tissue regeneration. Freeze-drying created the nanocomposite scaffolds. The effects of POSS inclusion on CS shape and structure were studied. Tests for cytocompatibility, cell proliferation, alkaline phosphatase activity, osteocalcin synthesis, and biomineralization were performed on the POSS nanoparticles. Osteoblast adhesion and proliferation, as well as alkaline phosphatase (ALP) activity, osteocalcin production, and cellular biomineralization, were all positively affected by POSS incorporation [21]. Cerium oxide nanoparticles (NC) are excellent free radical scavengers. By freeze-drying NC into gelatin–alginate scaffolds, nanocomposite scaffolds (GA-NCs) were created. It was also investigated how changing the nanoceria concentration affects the physicochemical and biological characteristics of the nanocomposites. The connected pores in the scaffolds were only detectable after a field emission scanning electron microscopy (FESEM) scan. The mechanical properties and biomineralization were enhanced by the addition of NC, while edema and in vitro weight loss were reduced. [22].

Graphene oxide and nanohydroxapatite (nHAp) have shown promise for bone tissue regeneration. This has led to increased studies into graphene oxide (GO) and nanohydroxyapatite (nHAp)-reinforced polymeric nanocomposite scaffolds. A three-dimensional porous polymeric nanocomposite scaffold made of gelatin–alginate (GA) and GO/nHAp was produced. Each component of the nHAp–GO/GA polymeric nanocomposite scaffold may work synergistically to promote tissue regeneration. The scaffold is physico-chemically sound [23]. The chitosan (CS)–hydroxyapatite (HA)–wollastonite (WS) bio-nanocomposite scaffolds were freeze-dried with 0, 10, 20, and 30% zirconium. The phase structure and morphology of the scaffolds were further studied using X-ray diffraction, SEM, and energy dispersive spectroscopy (EDS). The scaffolds were then tested for bioactivity and biodegradability. The bio-nanocomposite scaffolds absorbed water well due to their hydrophilic components and high-water absorption capacity. Prefabricated scaffolds showed no cytotoxicity across a wide concentration range of the scaffold extract. The investigated porous scaffold has promise in bone tissue engineering applications due to its similarity to the structure of actual bone [24]. A new biodegradable nanocomposite scaffold was created by crosslinking hydroxyethyl cellulose and soy protein isolate and adding montmorillonite. The prepared nanocomposites have improved mechanical and physical characteristics, which are ideal for bone tissue engineering during the breakdown of nanocomposites [25].

**Figure 2:**
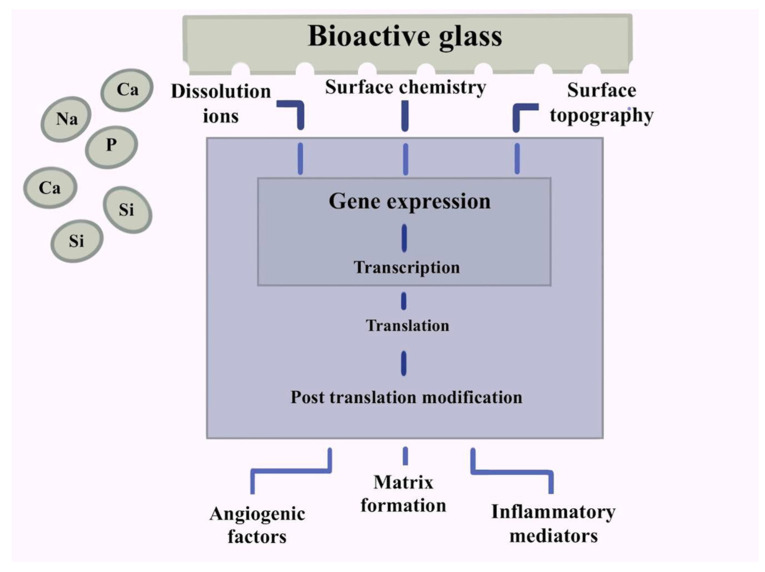
The structure of bioactive glass.

Nanocomposites were made using cerium oxide nanoparticles and scanned with an electron microscope, and spectroscopically and mechanically tested. The scaffolds’ pro-osteogenic character was assessed using a pre-osteoblast cell line’s adhesion, viability, and mineralization. Free radical scavenging (FRS) was assessed by measuring the breakdown of hydrogen peroxide and the cytotoxicity of cells on the scaffolds following oxidative stress induction. These studies confirmed the nanocomposites’ porosity, structural, and chemical characteristics. With no additional mineralization-inducing stimuli, the scaffolds demonstrated biocompatibility by improving cell adhesion, survival, and initiation [26]. Cells were combined with chitosan–gelatin polymers to create bone-healing bio-nanocomposite scaffolds. The scaffolds’ bioactivity was tested in simulated bodily fluids and with dental pulp stem cells. This study investigated the scaffold materials’ bone regeneration capabilities in vivo in rats. In vitro, the scaffold nanocomposites accelerated the crystallization of bone-like apatite. The alkaline phosphatase activity of bio-nanocomposites containing bioactive glass nanoparticles was increased [27]. Figure 2 shows the structure of bioactive glass. The figure shows that bioactive glasses have been shown to change gene expression in both hard and soft tissue repair. By changing the surface chemistry, topography, and release of dissolution ions, new resorbable bioactive glass constructs can change gene expression. This will make it possible to make tissue-specific scaffolds with specific surface chemistry, topography, and rate of ion release.

Free-radical polymerization produced a polymeric nanocomposite material for the porous nanocomposite scaffolds. The compressive strength (4.1 to 16.90 MPa), Young’s modulus (13.27 to 29.65 MPa), and pore size of these nanocomposites increased (from 63.72 1.9 to 45.75 6.7 m). Adding graphene oxide (GO) may regulate the porosity and mechanical properties of the nanocomposite scaffolds [28]. Nanocomposite interactions with human osteoblast cells and rat subcutaneous tissue were studied in vitro and in vivo. The scaffolds were biocompatible in all cases and improved cell adhesion, proliferation, mineralization, and infiltration. Hydrogen peroxide has been used to induce oxidative stress in osteoblast cells to test the nanocomposite scaffolds’ antioxidant capabilities. The scaffolds also exhibited biocompatible characteristics after in vivo implantation, which is required for effective bone tissue scaffolds. Cells could enter the scaffolds while the surrounding tissues generated a modest immune response. The creation of a nanocomposite scaffold system capable of supporting bone remodeling processes has been verified while protecting against free radicals [29]. Three-dimensional nanocomposite scaffold designs are being developed and tested to treat bone tissue replacement. Three-dimensional nanocomposite scaffolds created from them closely resemble bone tissue in shape, microstructure, and mechanical characteristics [30].

Poly (glycerol sebacate) (PGS) was another polymer investigated for its elastomeric characteristics. In a preliminary investigation, the cell adhesion of magnetic PGS nanocomposites with no surface characteristics was found to be poor. To decrease aggregation and improve dispersibility in polymer solutions to produce magnetic nanocomposites, different weight percentages of polyvinyl alcohol (PVA) were employed to alter the surface of superparamagnetic nanoparticles. When compared to the other groups, the 30 wt percent PVA coating provided the best dispersibility [31]. For bone marrow transplantation, substances such as hydroxyapatite (HA), which includes calcium and phosphorus ions, are required. Carbon nanotubes, like bone collagen, may be used to enhance the mechanical, chemical, and biological characteristics of nanocomposites.

**Figure 3:**
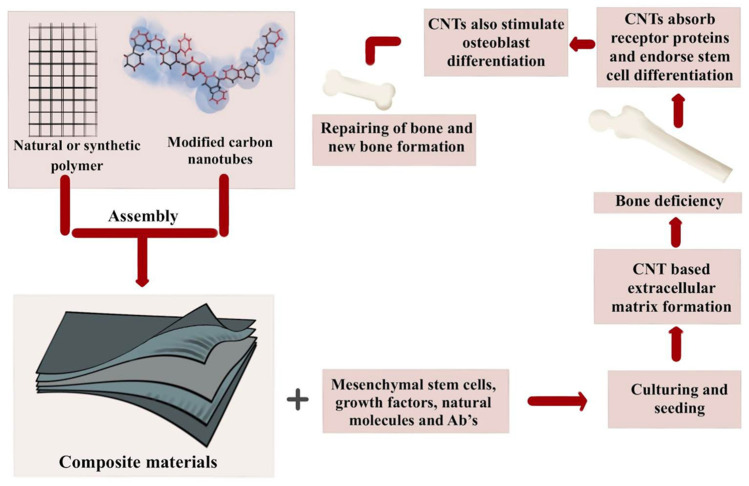
The fabrication process of carbon nanotubes and their roles in bone regeneration.

The development of a bio-nanocomposite scaffold that has all these characteristics is critical for speeding bone healing and minimizing the difficulties associated with existing scaffolding techniques. The results showed that the method, which included the digital light processing (DLP) approach, was an efficient strategy for developing novel scaffold bio-nanocomposites for use in bone tissue engineering [32]. The fabrication process of carbon nanotubes and their roles in bone regeneration are shown in Figure 3. The figure shows that there are no natural or synthetic biomaterials that can be used to make bones that look and work like real bones under normal conditions. Carbon nanotubes (CNTs) have unique properties that make them a good candidate for making new biomimetic materials in the bone biomedical field. It is true that CNT-based materials and their composites could have a major impact on the design and integration of bone scaffolds or implants, as well as in drug therapeutic systems.

The mechanical characteristics and apatite generation ability of synthetic fluorapatite-hardystonite (FA-HT) nanocomposite scaffolds were investigated. Hardystonite (HT; 5 and 10% by weight) was used as a reinforcement phase in the FA scaffold. FA and HT were mixed for 4 h at 220 °C under argon gas. Porous FA-HT scaffolds were created using a space holder technique. The holes in this technique were created using sodium chloride (NaCl). The powder was then crushed at 220 MPa pressure. Finally, the samples were sintered for 2 h at 1000 degrees Celsius. The results of X-ray diffraction (XRD) on the fabricated scaffolds validated the synthesis of FA and HT powders. The Young’s modulus values of these scaffolds at 12.4 MPa and 5.5 MPa were also above average. The scaffolds’ Ca/P ratio was 1.71 ± 0.3 for the FA-5HT sample and 1.60 ± 0.5 for the FA-10HT sample, as determined by the bioactivity test. The results indicate that FA-HT scaffolds, with their favorable mechanical properties and adequate level of bioactivity, are novel and promising biomaterials for bone tissue engineering and the repair of bone defects [33]. The shape, structure, mechanical performance, and release behavior of this nanocomposite scaffold were investigated for scaffold characterization. The nanocomposite scaffolds were investigated in cells using the 3-[4,5-dimethylthiazole-2-yl]-2,5-diphenyltetrazolium bromide (MTT), alkaline phosphatase (ALP), and calcium deposition assays. The osteogenesis and release characteristics of the 2-GHPr nanocomposite scaffold were the best of all the scaffolds examined. With regards to osteogenesis and releasing behavior, the 2-GHPr nanocomposite scaffold was superior to the other manufactured scaffolds. Out of all the scaffold types evaluated, 2-GHPr scaffolds had the highest mechanical strength and modulus. Bone tissue engineers have been discussing the potential of 2-GHPr composite scaffolds [34].

The free radical polymerization technique was used to create a nanocomposite using acrylic acid (AAc), guar gum (GG), nano-hydroxyapatite (HAp NPs), titanium nanoparticles (TiO_2_ NPs), and the optimal graphene oxide (GO) quantity. The scaffolds were made by freezing them and covering them with silver sulphadiazine. The functional group, crystal structure characteristics, morphology, elemental properties, porosity, and mechanical properties of the produced scaffolds were studied using various methods [35]. It produced bioactive nanocomposite scaffolds composed of poly (caprolactone) and bio-active glass nanoparticles with a highly spaced and fibrous network macrostructure. The scaffolds were readily shaped and hydrophilic to quickly absorb water and blood. An osteogenic cell line was created by infiltrating multipotent stem cells from tooth pulp into the scaffold networks and stimulating them to proliferate and differentiate into osteogenic cells. The cell/scaffold constructions fitted irregular-shaped alveolar bone defects and stimulated early new bone growth. With multipotent dental stem cells, electroblown bioactive fibrous scaffolds may be used as a future three-dimensional platform for customized bone tissue creation [36].

The composite scaffolds’ morphology, diameter, components, hydrophilicity, and biodegradability were studied. In vitro proliferation, differentiation, and mineralization of cells on various nano-fibrous scaffolds were studied [37]. Healing of load-bearing segmental lesions in long bones was difficult owing to complicated weight distribution and bending, shearing, axial, and torsional stresses. Its biomechanical properties were developed to resist cortical bone strength. Freeze drying and adding copolymers to the materials created an optimum surface permeability for encouraging bone formation [38]. The porous scaffold underwent static and dynamic stress conditions. An isotropic linear material was used to simulate the maxillofacial bone using the Solidworks program [39]. Hybrid material analysis and preliminary mechanical characterization. This class of hybrid materials has great promise as active biomechanical bones for growing osteoblasts and differentiating stem cells. These hybrid nanocomposites have significantly greater mechanical strength than the hydrogels used for bone regeneration and could be used as an osteoinductive covering for metal trabecular scaffolds. These hybrid nanocomposites can be employed as an osteoinductive coating for metal trabecular scaffolds, compensating for the shortcomings of hydrogels in bone regeneration. The normal macro- and micro-distribution of stresses and deformations in bone are thought to be mimicked by micro-trabecular metal structures coated with active and osteoinductive biomechanical ceramic-polymeric biomechanical scaffolds [40].

The degradation of titanium dioxide (TiO_2_) nanoparticles and nanocomposite scaffolds is being investigated. In vitro testing included using bladder tumor (UC6) and osteosarcoma (MG-63) cell lines. The TiO_2_ nanoparticles and polymeric nanocomposites’ efficacy against *E. coli* and *S. aureus* and their in vitro biocompatibility has been shown. These results suggest that polymeric nanocomposites and TiO_2_ nanoparticles are both viable options for usage in biomedical settings due to their desired physicochemical and mechanical properties. It has been reported that a biomimetic chitosan–sodium alginate scaffold containing TiO_2_ nanoparticles (1 wt%) has improved biocompatibility for use in bone tissue engineering [41]. Polylactic acid (PLA) nanocomposite scaffolds containing magnetic and conductive fillers were investigated for bioactivity and degradation. The bulk porosity of the porous structures that were 3D printed was found to be 50%. In vitro tests showed that the bioactivity of scaffolds increase by anywhere from 2.9% (PLA) to 5.3% (PLA/CNF) and 3.12% (PLA/Fe_2_O_3_). Based on the results of the compression tests, it was found that the PLA composite was less rigid than expected, with a rigidity of 533 MPa (PLA/CNF) and 425 MPa (PLA/Fe_2_O_3_).

PLA nanocomposites with conductive fillers are attractive as scaffolds for bone replacement and regeneration in tissue engineering because they are more bioactive and break down quickly [42]. Fourier-transform infrared spectroscopy (FTIR), scanning electron microscopy (SEM), and X-ray diffraction were used to analyze the scaffolds’ functional groups and surface morphology. The samples of BNS showed a substantial antibacterial effect against DH5-alpha *E. coli.* Experiments using the MC3T3-E1 cell line and a neutral red dye assay demonstrated that the scaffolds were non-cytotoxic and biocompatible. There are a wide variety of applications for these bioactive scaffolds in bone tissue repair and regeneration [43]. Antibacterial copper, bronze, and silver nanocomposites were produced using fused filament fabrication (FFF). The increases in both antibacterial properties (20–25%) and bioactivity (18–100%) can be attributed to the acid treatment and reinforcing metallic/metallic alloy particles [44]. In addition to examining the scaffolds’ thermo-mechanical characteristics, bone is a hierarchical nanomaterial made up of organic (collagen) and inorganic (nano-hydroxyapatite). The goal is to regenerate and mend bone tissue using scaffolds. Regeneration of bone tissue takes place when biopolymer nanocomposites that resemble the bone’s native architecture regulate cell proliferation, differentiation, and migration [45].

**Figure 4:**
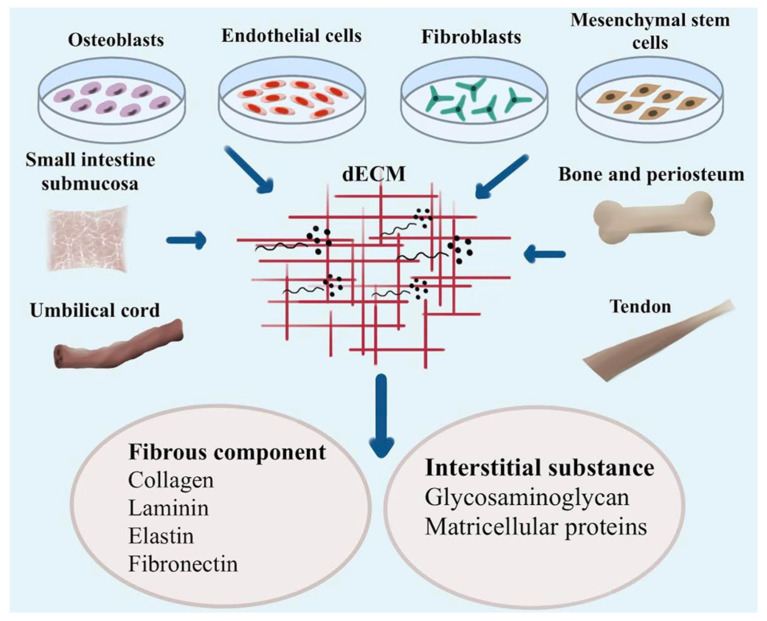
The fibrous component during the process of bone regeneration.

Both nanocomposite scaffolds were antibacterial against *P. aeruginosa*, *S. aureus*, *B. cereus*, and *E. coli*. Promising fluid absorption, biodegradability, and antimicrobial activity suggest their biological potential. According to the biodegradability test, semi-IPN and IPN will decompose by 73 and 61% in garden soil and 75 and 64% in bio-compost, respectively. Average particle size was determined to be 11.3 nm for Ag0/Bs-cl-polyAAm-Gm and 8.6 nm for Ag0/Bs-cl-polyAAm-IPN-AA-Gm [46]. The fibrous component during the process of bone regeneration is shown in Figure 4. The figure shows that MSCs may be found in adipose tissue, muscle, tendons, and peripheral blood vessels. Cells that originate from bone marrow, periosteum, and vessel walls are known as stromal stem cells (MSCs). There is a complex interplay of chemicals, cells, and metabolic processes in bone tissue while it develops and forms. Factors including sufficient MSCs and the surrounding environment are critical for fracture healing.

Dexamethasone and simvastatin were added to electrospun nanocomposite polycaprolactone (PCL) scaffolds. FTIR was used to find out about the scaffolds by measuring their wetness, pH, and drug release. Electron microscopy showed that GO was spread out evenly across PCL nanofibers that were 1 nm or less in thickness. In comparison to PCL scaffolds, adding GO and drugs improved the hydrophilicity, cell survival, osteogenic differentiation, and pH. Even though cell survival was reduced in the ALP experiment, the PCL/GO–Dex scaffolds showed a lot of differentiation [47]. Because of their enhanced biocompatibility, alkaline phosphatase activity, and calcium deposits, manufactured nanocomposite scaffolds may be osteoinductive materials for bone repair and regeneration. The findings demonstrate that the scaffold with graphene oxide (GO) exhibited enhanced levels of biocompatibility, alkaline phosphatase activity, and calcium deposits, thereby emphasizing the hypothesis that fabricated nanocomposite scaffolds are promising osteoinductive products for bone repair and regeneration [48]. Gelatin/bioactive glass (BG–Gel) nanocomposite scaffolds were reinforced with cellulose nanocrystals (CNC). In situ composite and freeze-drying produced BG–Gel–CNC. CNC improved the wettability and compressive strength of the scaffolds, which in turn increased cell adhesion, growth, and proliferation above the control. All the parameters for biocompatibility, pore connectivity, and porosity were satisfied [49].

## 3. Nanocomposite Ceramic’s Potential Role in Bone Regeneration

In bone graft replacement, nano-hydroxyapatite is extensively utilized due to its biocompatibility and osteoconductive characteristics. Ceramic, metal, and polymer hydroxyapatite nanocomposites have been used in biomedical areas, including bone tissue engineering. Nanocomposite materials and drug delivery systems can now incorporate a wide range of properties [50]. Morphology is important in bone healing and regeneration. It is ideal for biological system activity and biocompatibility because it is ideal for bone scaffold production. Ultrasonication was used to create ultra-long tricalcium phosphate (UTCP). The UTCP scaffold was reinforced with methacrylate chitosan (MAC) polymer. The results provide further evidence that the composite is a conducive environment for healthy cell growth. The results of in vivo and clinical research indicate that it has potential as a bone implantation preparation and as an aid to the speedy regeneration of bone tissue [51]. The effects of tricalcium phosphate (TCP) on nanocomposites’ shape, crystalline structure, functional groups, and thermal behavior were studied. SEM was used to examine ultrathin cross-sections of the nanocomposites, which revealed that all except the polycaprolactone (PCL)-TCP fibers had an average fiber diameter (AFD) of about 100 nm [52]. For bone tissue engineering, metallic implants such as magnesium-based alloy foams may be utilized as a potential scaffold material due to their mechanical strength. Magnesium foams are also biocompatible and biodegradable, allowing for no need for revision surgery following implantation in orthopedic applications [53]. The microstructures of these nano-ceramic reinforced metal matrix foams were investigated using SEM, energy dispersive X-ray spectroscopy, X-ray diffraction, and X-ray micro computed tomography (X-ray micro-CT) [54]. Nanocomposites are composites made of ceramic, metallic, or polymeric materials. This allows for the incorporation of many characteristics into nanocomposite materials, such as magnetism, Magnetic Resonance Imaging (MRI) contrast, and drug delivery. In vitro cell investigations showed that the biocomposites were effective in regenerative settings [55].

The robocasting core-shell bioceramic scaffolds were created and tested in an alveolar bone defect model in beagles. The Mg-doped calcium silicate (CSi-Mg5) shell easily contributed to the initial mechanical strength and early-stage osteogenic activity of the TCP@CSi-Mg5 scaffolds, including adjustable ion release, improved biodegradation, and excellent osteogenesis capacity in alveolar bone defects [56].

The antibacterial efficacy of a glass–ceramic material containing strontium ions (Sr^2+^) against oral infections was also evaluated. Using the sol-gel method, a glass–ceramic bioactive material in powder (CP) was created based on S53P4 bioactive glass. The results showed that higher concentrations of Sr^2+^-doped CP materials can improve bone healing and regeneration in lesions of critical size [57]. Porous biphasic calcium phosphate (BCP) ceramic spheres with nanocrystals (BCP-N) were created by combining alginate gelatinizing and microwave hybrid sintering methods. BCP granules with microcrystalline (BCP-G) and commercially irregular (BAM, BCP-I) characteristics were used as controls. BCP-N is highly effective in directing bone regeneration and shows promise as an alternative to traditional bone grafts for use in filling bone defects [58]. These scaffolds were orthotopically implanted in experimental rat models to evaluate their capacity to repair an induced bone deficiency. Within five weeks, using magnetic resonance imaging and histological analysis of processed samples, new bone tissue was observed to be growing around the scaffolds as the regeneration process progressed [59]. The phase inversion technique was utilized to create polymer–ceramic film structures. This required depositing a composite solution on a glass support and scaling it to a 0.2 mm thickness. The produced composite film structures were verified as effective methods for directed bone regeneration [60] After thermal treatment, the bioglasses were three-dimensionally printed into porous scaffolds, and the silicon dioxide (SiO_2_)/calcium oxide (CaO) molar ratio was changed from 90/5 to 60/35. Three-dimensionally printed scaffolds have a linked porous structure with adjustable porosities. The microstructure, deterioration, ion dissolution, and apatite production were studied. High SiO_2_ concentrations resulted in more uniform crystalline particles and sintering compactness, which resulted in increased strength [61]. In vitro, the CaO–Magnesium oxide (MgO)–SiO_2_-based bioactive glass–ceramic coating (named M2) on a Ti–6Al–4V alloy performed well. Compared to the commercially available HA-coated Ti-6Al-4V implant, the M2-coated implant showed promising results in repairing load-bearing bone defects [62]. Three-dimensional bio-plotting, an RP technique, was used to construct scaffolds from pure poly-caprolactone (PCL) and a PCL/ceramic micro-powder hybrid.

A cellular lattice structure was created by utilizing a 0/90° laydown pattern with a continuous contour filament to create interconnected porous reticular structures. The results provide credence to the utilization of 3D bioplotted PCL/bovine bone filler Nukbone (NKB) scaffolds in a wide range of tissue engineering applications, particularly for the promotion of bone tissue regeneration [63]. For bone tissue engineering, a series of nanocomposite scaffolds with dextran (Dex) and sol-gel-generated bioactive glass ceramic nanoparticles (nBGC: 0–16 wt%) were created. SEM revealed that the Dex/nBGC scaffolds had a porous, three-dimensional microstructure with an average pore size of 240. At low nBGC concentrations (2 wt%), nanoparticle distribution was homogeneous inside the Dex matrix, whereas agglomeration was seen at higher levels [64]. Particle size distribution and Fourier-transform infrared spectroscopy were used to characterize the polymer-coated drug ceramic nanocomposite (DOX-HAp-PVA). The DOX-HAp-PVA nanocomposite showed significant cytotoxicity against osteosarcoma cells (MG 63), suggesting it might be utilized as an osteosarcoma anticancer agent [65]. Dental implants constructed of a novel ceramic nanocomposite composed of alumina and ceria-stabilized TZP (ZCe-A) were utilized to assess bone and soft tissue integration in a dog model. The results demonstrated the clinical viability of an Al_2_O_3_/Ce-TZP nanocomposite, and the superior mechanical qualities obtained by this material position it as an enhanced alternative to conventional 3Y-TZP dental implants [66]. Electrophoretic deposition (EPD) is a simple, quick, and low-cost way to create homogeneous coatings. Coating polymer–ceramic composites requires EPD, utilizing binders that do not require heat degreasing, which also removes the polymer components. A unified electrophoretic deposition (EPD) coating of a bone-like hydroxyapatite/collagen nanocomposite (HAp/Col) nanocomposite on a titanium (Ti) substrate has been demonstrated. Varying the applied voltage and/or treatment duration effectively regulated coating thickness. The modified EPD coating’s tape test adhesive strength was class zero compared to the non-Mg^2+^ EPD coating’s class five [67].

A tri-layered nanocomposite hydrogel scaffold, with or without growth factors, was implanted into rabbit maxillary periodontal defects. The tri-layered nanocomposite hydrogel scaffold with growth factors is an alternative regenerative approach that can enable simultaneous and complete periodontal regeneration [68]. Combining hydroxyapatite/poly (vinyl alcohol) nanocomposite coatings with porous Composite composites with TiO_2_ ceramic show improved mechanical characteristics and in vitro bioactivity. Combining hydroxyapatite/poly (vinyl alcohol) nanocomposite coatings with porous TiO_2_ ceramic resulted in a composite material with enhanced mechanical characteristics. There was an increase in in vitro bioactivity and an initial mechanical strength of up to 0.99 ± 0.19 MPa as a result of this combination [69]. Because of their comparable structure and high biocompatibility, ceramic structures are frequently used in bone and tooth regeneration. Because hydroxyapatite is biocompatible, bioactive, and bioresorbable, it is used to repair bone and teeth. Gelatin has outstanding emulsifying, film-forming, and water-binding characteristics [70]. Nanostructures with homogeneous ceramic particle can disperse in CS polymers. Apatite particles are precipitated by immersion in corrected simulated body fluids (C-SBF) at 37 °C. In vitro bioactivity, biomineralization, and cell attachment showed that all coatings had apatite-induced abilities and cell compatibility [71].

Synthetic bone replacements have long struggled to develop porous polymer host matrices loaded with bioactive ceramic particles capable of initiating cellular organism reproduction while maintaining in vivo mechanical dependability. A mechanochemically robust, crosslinked aromatic backbone was formed using endothermic condensation polymerization [72,73]. The nanocomposites were made up of metallic Fe nanoparticles dispersed in a porous ceramic matrix made up of amorphous silica and alumina. The magnetic properties of these nanocomposites were investigated. Experiments verifying DNA separation viability proved the produced materials’ viability [74]. With the active involvement of the surrounding tissues, particularly blood vessel invasion into the implant, the nanocomposite metal-ceramic implant demonstrated excellent efficacy for the replacement of bone defects and regeneration. Nanocomposite metal ceramic for large-flap craniotomies and delayed cranioplasty has been shown to be effective in preclinical testing utilizing rat bucks [75].

A three-dimensionally printed femur with a middle hip composed of a polylactic acid–hydroxyapatite nanocomposite with 0, 5, 10, 15, and 25% ceramic nanoparticles was loaded in three ways: centrally, fully, and partly. Recent developments in 3D printing technology include the widespread availability of high-temperature, material-extruding, thermoplastic printers, composites, binder jetting for metal, and the merging of existing businesses. In addition, the capacity to handle a wider range of innovative materials will be crucial for the next generation of printers, notably industrial-grade solutions [76]. Freeze-drying has been used to examine the impact of different elastin biopolymer quantities on porous bio-nanocomposite scaffolds. The biological properties of the sample showed that apatite forms a thick layer on the surface, while the alkaline group increased at a stable pH concentration. The measured porosity and elastic modulus were used to determine if a given micromechanical model was likely to work. When comparing several micromechanical models of the porous bone substitute, error rates of less than 10% were found [77]. The magnetic behavior of ceramic nanocomposites made from transition metal-laden zeolite precursors was addressed, as was their potential in biomedicine and pollution remediation. This is because the magnetic behavior of ceramic nanocomposites derived from transition metal-loaded zeolite precursors is exceedingly complex due to the presence of both zero-valent transition metal nanoparticles and transition metal ions dissolved in the matrix [78]. The structure of biological composites like nacre and bone with high filler (ceramic) content results in great strength and toughness. A multifunctional bio-nanocomposite has been created with strength, toughness, and corrosion resistance. The results showed that multilayer construction with a convoluted diffusion route improved corrosion resistance [79].

## 4. Contribution of Nanostructured Biomaterials to Bone Regeneration

Biomaterials should stimulate bone tissue regeneration and ultimately disintegrate in situ to be replaced by the newly produced bone tissue. This field aims to develop biomaterials that promote bone regeneration and vascularization [80]. Using biodegradable polymer-based biomaterials facilitates ordered cell and tissue development and repair by providing structural support and biological confinement. The capacity of natural polymers to adapt to the application location is employed in bone tissue engineering. It is also employed in bone tissue regeneration Using polymers such as poly (lactic acid), poly (lactic-co-glycolide), and polycaprolactone. Tissue engineering applications of nanocomposites comprised of biodegradable polymers were investigated and utilized recently [81]. The use of biodegradable polymers to create composites that combine flexibility with stiffness and bioactivity has opened new possibilities. Synthetic and natural polymers have been successfully mixed with BG nanoparticles and nanofibers. BG–polymer nanocomposites are more like genuine bone [82]. To design new biomaterials with specific properties, computer simulations such as molecular dynamics, quasi-classical spin dynamics, Langevin’s and Boltzmann’s equations, and Monte Carlo simulations have been developed.

The impact of blood flow at 310.15 K was studied in polyurethane/graphene nanocomposites, which absorb polymethyl methacrylate and calcium carbonate, respectively, and generate calcium phosphate [83] in situ during the growth of CaP nanoparticles (ICPNs) during free-radical polymerization of the DMAEMA and HEMA matrix (PDH) for bone regeneration. Carboxyl-Ca^2+^ coordination and subsequent CaP precipitation generate ICPNs via the inclusion of poly-l-glutamic acid (PGA) with numerous carboxyl functional groups. PGA carboxyl groups may also interact with dimethylaminoethyl methacrylate (DMAEMA) tertiary amines to increase hydrogel mechanical strength [84]. Traditional nanoparticles’ high surface energy contributes to aggregation and heterogeneity, causing bone deformity in orthopedic surgery. In vitro, carboxyl-functionalized synthetic polymers resemble non-collagenous proteins’ carboxyl-rich surface motifs in stabilizing hydroxyapatite and driving intrafibrillar mineralization. Using a biomimetic method, carboxyl-functionalized poly (lactic-co-glycolic acid) may achieve high material homogeneity. This approach improves the mechanical characteristics of nanocomposites and optimizes controlled drug release, resulting in improved cell growth and osteogenic differentiation [85].

It offers improved mechanical strength, electrical conductivity, and continuous phosphate ion release. The cross-linking matrix was biodegradable oligo (poly (ethylene glycol) fumarate (OPF), with cross-linkable CNT-poly (ethylene glycol)-acrylate (CNTpega) for mechanical support and electrical conductivity. Two-dimensional (2D) black phosphorus nanosheets were also injected to help regenerate tissue by releasing phosphate from ambient phosphorus oxidation in situ [86]. A self-healing Au-based 4-arm thiol terminated poly (ethylene glycol) [Au-(PEGSH)4] dynamic hydrogel was combined with 100 nm bioactive glass (BAG) nanoparticles agglomerated into 10 m clusters to form hydro-gel nanocomposites [Au-(PEGSH)4–BAG]. Using a double barrel syringe, an aqueous solution of a (PEGSH)4 homopolymer containing varying quantities of BAG nanoparticles was concurrently injected with an aqueous solution of chloroauric acid (HAuCl4) [87].

**Figure 5:**
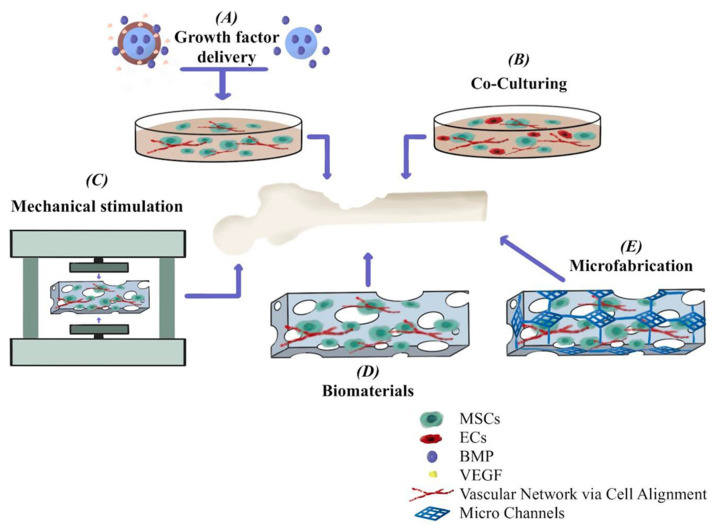
Bone regeneration process with the contribution of biomaterials.

A gelatin–chitosan polymeric membrane with hydroxyapatite and titania nanoparticles has been developed, both of which are osteoconductive materials. The nanocomposite membrane was cross-linked using non-toxic ultraviolet (UV) radiation to increase its thermophysical and mechanical properties and manage its biodegradability. Cell adhesion and proliferation experiments were used to assess the novel nanocomposite’s in vitro biocompatibility. Using mouse embryonic fibroblast (MEF) cells, osteoconductivity was assessed [88]. The bone regeneration process with the contribution of biomaterials is shown in Figure 5. The figure shows that different methods have been used to induce vascular networks formation in engineered constructs for bone regeneration. These include providing growth factors, using coculturing systems, applying mechanical stimulation, using biomaterials with the right properties, and using microfabrication techniques.

For the application of bone tissue regeneration, the produced composite Hydroxyapatite (HAP)/poly xylitol sebacic adipate (PXSA)/K (VK) was examined. The produced composites were characterized using FTIR, X-ray diffraction, SEM, and TEM. UV–vis spectroscopy confirmed the release of VK from the HAP/PXSA/VK combination. The HAP/PXSA/VK composite was suitable for MSC culture in vitro. The HAP/PXSA/VK composite had better microstructures and biodegradation characteristics than pure HAP synthesized using the same procedure [89]. The objective of regenerative medicine is to create synthetic biomaterials that have the inorganic and organic properties of real bone. Due to its inherent bioactivity and osteoconductivity, calcium phosphate has been used to simulate the inorganic components of bone, such as calcium hydroxyapatite [90].

These films include chitosan, polyvinyl alcohol, graphene oxide, hydroxyapatite, and gold. The graphene oxide/hydroxyapatite/gold (GO/HAP/Au) nanocomposite was synthesized by a simple hydrothermal process, and the polymeric film was made by the gel casting method. The nanocomposite was studied by XRD, HR-TEM, FE-SEM, and FT-IR. Less alkaline phosphatase activity in the cells indicated that the biofilms were biocompatible with mouse mesenchymal cells [91]. The bilayer membrane’s top layer inhibited epithelial and fibroblastic cell migration and proliferation, whereas the sub-layer promoted osteogenic cell bioactivity at the defective location. Freeze-drying and electrospinning were used to create microporous and nanofiber layers. An enzymatic degradation study was performed on the composites to determine their morphological, mechanical, and physical characteristics. In vitro, the antimicrobial properties of the nanocomposite membranes were investigated [92].

An in vivo investigation was performed on the chitosan–nano-hydroxyapatite (nHA) composite for bone tissue regeneration. The use of chitosan–nHA composites with third-component systems (synthetic polymers, growth agents, and stem cells) was studied. The chitosan–nHA composite was shown to be a potential biomaterial for bone tissue engineering [93]. The biomaterials’ physicochemical characteristics regulate osteoblast proliferation and differentiation. Inspired by natural bone’s electrical characteristics, electroactive composites for osteogenesis have increasingly become a research hotspot. There is a structure–activity link between electrical properties, specific surface potential, and osteogenic activity a study using an electroactive poly (lactic-co-glycolic acid) biocomposite containing gadolinium-doped barium titanate nanoparticles [94]. Biomaterials are used to treat and improve many tissues and organs. Metallic, organic, and composite materials are now used. Metallic materials have excellent mechanical strength and corrosion resistance, whereas organic polymeric materials are biocompatible, biodegradable, and naturally available. Coatings are often used to improve the biocompatibility of certain metals and alloys. This is due to their outstanding biocompatibility and biodegradability. Hydroxyapatite (HAp) is a ceramic substance that may be used to coat metals for biomedical purposes [95].

Using small interfering RNA (siRNA)-decorated particles, researchers created a nanostructure covering on titanium implants for synergistic skeletal and vascular tissue regeneration. The siRNA was coupled to nanoparticles to target cathepsin K regulation. The functionalized nanoparticles formed a hierarchical nanostructured coating on the bone implant. By controlling messenger RNA (mRNA) transcription, the coating promoted cell survival and growth factor release [96]. Studies on rat calvaria indicate that nanostructured carbonated hydroxyapatite microspheres (nCHA) are an efficient alternative to collagen membranes for recombinant human bone morphogenetic protein-2 (rhBMP-2) distribution [97]. Nanocrystalline silicate-substituted hydroxyapatites (nSi-HAps) containing Eu^3+^-doped metal molybdates were functionalized with bismuth (3+) ions. Microwave-assisted hydrothermal biomaterials were produced and heat treated at 700 °C. The concentration of Eu^3+^ ions was 1 mol%, while Bi^3+^ was 0.5–2 mol%. The biomaterials’ physicochemical characteristics were evaluated using standard methods such as X-ray powder diffraction, scanning electron microscopy, and infrared spectroscopy. In this study, the Rietveld technique [98] was used to determine the diameters of the particles, which ranged from 22 to 65 nm. A stimulation of patterned biomaterials seeded with stem cells was not tested. On solid and nonporous micropyramid-patterned Si surfaces, the impact of electrical stimulation on osteogenic differentiation of rat bone marrow-derived mesenchymal stem cells was studied. Both stimulation and scaffold patterning improved osteodifferentiation. The stimulated nanoporous micropyramid scaffolds outperformed the stimulated solid micropyramid surfaces in promoting rat bone marrow mesenchymal stem cells’ (rBMSCs’) osteogenic differentiation [99].

To illustrate naturally graded systems, engineers and researchers have exploited teeth and bones to design unique bioinspired solutions [100]. These studies looked at human bone mesenchymal stem cells (bMSCs) cultivated on titanium dioxide (TiO_2_) nanotubular surfaces and their osteoclastogenesis-related cytokine expression in conditioned medium (CM) produced by macrophages cultured on the same surfaces (NT-CM) [101]. Tissue engineering and regenerative medicine are now focusing on the repair of bone and dental injuries and disorders. These rigid tissues may be repaired using biodegradable materials. Among other materials, nanostructured biomaterials have shown promise. These nanoengineered biomaterials outperform traditional materials in the creation of bone and dental tissue. The surfaces of nano-scale components are also conducive to cell interactions [102].

Insufficient integration into host tissue, inflammatory responses, and infection can restrict the performance of orthopedic implants. The surface features of these bone biomaterials influence the biological activities of immune and osteogenic cells. Modern nanofabrication techniques have enabled us to create nanostructured surfaces with regulated physicochemical qualities that influence osteogenesis-related and immune cell activity, affecting bone integration and local immune response. This article summarizes the development of nanostructured surface modifications to bone implants with controlled physicochemical characteristics [103]. Current biomaterials are being tested for their capacity to tolerate both host tissue cell responses and bacterial contamination. The antibacterial properties of biocompatible Mg^2+^-substituted nanostructured hydroxyapatite (HA) was studied. *S. aureus*, *P. aeruginosa*, and *E. coli* densities were considerably reduced following growth in Mg-substituted HA materials, indicating a Mg^2+^-Ca^2+^ transition in the HA lattice [104].

## 5. Discussion

Bone fractures are by far the most common kind of traumatic injury that may occur. It is a regenerative process that happens throughout the period of embryonic skeletal development, and it is responsible for the repetition of many of the biological processes that occur during that period. This is shown by the breaking of a bone. A successful healing process as well as the restoration of structural integrity and strength to a shattered bone are usually the invariable consequences of this procedure. When a person experiences a fracture, the body goes through a process known as bone regeneration to rebuild the broken bone. Bone development is a difficult and painstakingly organized physiological process that takes time and effort. Additionally, as an adult, it undergoes a continuous remodeling process, which may be seen during the normal healing period of a fracture.

It is becoming more vital in the process of bone regeneration to use polymer nanocomposites, which are composites made up of a polymer matrix and an additional nanomaterial. This paper discusses the contributions made to the progress of the field by the most recent advancements in polymer nanocomposites, which are being used in the process of bone regeneration. The use of nanocomposite scaffolds, nanocomposite ceramics, and other types of biomaterials in bone regeneration is currently being researched. Recent breakthroughs in polymer nanocomposites may be used in a range of industrial processes in the future, which will be advantageous to those who suffer from bone abnormalities today.

The impact of varying the concentration of nanoceria in nanocomposites on the physicochemical and biological properties of the nanocomposites was also investigated. In this study, a unique biodegradable nanocomposite scaffold was created by crosslinking hydroxyethyl cellulose/soy protein isolate and adding montmorillonite to a previously discovered biodegradable nanocomposite scaffold. The improved mechanical and physical properties of the nanocomposites that have recently been developed make them especially well-suited for the creation of bone tissue in humans. During the breakdown of nanocomposites, calcium and magnesium ions are released in a controlled manner, and these ions are important signals in the process of bone repair and regeneration. It was discovered and proven that the required porosity, structural, and chemical features of the nanocomposites, as well as their composition, were all present in the samples tested. When cells adhered to scaffolds, they survived longer and were more likely to initiate new growth. This reduced or eliminated the requirement for further mineralization-inducing stimuli. Using simulated bodily fluids and dental pulp stem cells, the scaffolds were evaluated for bioactivity, allowing the researchers to establish their efficacy and usefulness. In this study, the bone regeneration capabilities of the scaffold materials were evaluated in vivo rather than in the laboratory, and rats were used as the test subjects.

It was previously shown in the laboratory that scaffold nanocomposites aided in the crystallization of bone-like apatite in vitro, which was demonstrated by the researchers. A minimal immune response was seen in the surrounding tissues, even though cells were able to penetrate the scaffolds. According to the results, a nanocomposite scaffold system capable of supporting bone remodeling processes while simultaneously offering protection against free radicals has been shown to be viable and effective in the surrounding tissues, even though cells were able to penetrate the scaffolds. Nanocomposites containing hyaluronic acid, which are constructed of ceramic, metal, and polymers, have been successfully used in a range of biological applications, including bone tissue regeneration and bone graft regeneration, amongst others. Nanocomposite materials and drug delivery systems presently have the possibility of combining a wide variety of characteristics into their construction. This functionality is already available to users. The potential for mechanically strong metallic implants, such as magnesium-based alloy foams, to be used as prospective scaffold materials for bone tissue engineering has led to an investigation of their usage as scaffold materials for bone tissue engineering. Because of the biocompatibility and biodegradability of magnesium foams in orthopedic applications, the need for re-implanting magnesium foams is reduced once the first implantation of the foam is complete.

This study showed that a nanocomposite metal–ceramic implant was successful in the repair of bone defects and the regeneration of bone when the implant was actively engaged by the surrounding tissues, particularly when blood vessels invaded the implant’s interior cavity. Because of their propensity to disintegrate in the presence of oxygen, using biomaterials in conjunction with bone tissue regeneration offers the benefit of allowing for the replacement of old bone tissue with new bone tissue. As a result of this research, biomaterials that will help with bone regeneration and vascularization, among other things, will be developed. In recent years, researchers have investigated and exploited nanocomposites consisting of biodegradable polymers in tissue engineering applications, and it is expected that this trend will continue in the future as well. Due to the increasing usage of biodegradable polymers in the fabrication of composites that combine flexibility with stiffness and bioactivity in recent years, an astonishingly diverse spectrum of new applications has opened for scientists across the globe to explore. Several manufactured and naturally occurring polymers have been found to work well when mixed with BG nanoparticles and nanofibers, resulting in exceptional outcomes in the laboratory. BG–polymer nanocomposites have a more natural appearance than actual bone material, which is due to the utilization of polymer nanotechnology in the manufacturing process. In their current state of development, the technologies used in the bone regeneration process are still in the early phases of development, and it is predicted that as the field continues to advance, new and innovative technologies will emerge. Table 1 shows the basic principles, advantages, and limitations of three different types of nanocomposites for bone regeneration.

## 6. Conclusions

A wide range of physiological factors have an impact on bone formation. For bone regeneration therapies to be successful, drug delivery mechanisms and cell differentiation carriers must be used in conjunction. Polymers have recently acquired popularity because of their rapid localized absorption and ability to stimulate autologous bone repair in the body. Tissue engineering and other technological advancements are helping to meet the massive therapeutic need for bone autograft substitutes that currently exists. It is critical to employ biodegradable matrices that preserve cells while also promoting tissue development until the transplantation procedure is complete. Their biocompatibility and bioactivity are often superior to polymers in terms of performance. The effect of polymer nanocomposites on bone regeneration was examined in this review article. Our discussion focused on three distinct forms of polymer nanocomposites: nanocomposite scaffolds, nanocomposite ceramics, and biomaterials. Their interactions have been examined in the context of bone therapy. It is now being explored if there are any novel approaches to bone regeneration that may be used to hasten the healing process.

## Figures and Tables

**Table 1:** The basic principles, advantages, and limitations of different types of nanocomposites for bone regeneration.

Nanocomposites	Function	Advantages	Limitations
Scaffolds	A scaffold is a unique vehicle for transporting cells and drugs. In the field of regenerative medicine, scaffolds serve as biological mediums that promote cell proliferation and differentiation.	Scaffolds aid in the healing of cutaneous wounds by encouraging the differentiation of endothelial and epithelial cells and the production of angiogenic growth factors. Their biocompatibility ensures that they will not trigger any sort of immune response.	To spin the compositions, it is necessary to find the optimum solvent ratio. Fail to contain functional groups essential for protein binding or cellular adhesion.
Ceramic	Many metalloid solids, such as oxides, carbides, carbonates, and phosphates, can be produced by heating to a high temperature and then rapidly cooling. In addition, they contain both metallic and nonmetallic elements, as well as oxides, carbides, and nitrides.	In the fight against cancer and other diseases, including bacterial infections and glaucoma, they have been used as drug delivery systems. Even at nanoscale thicknesses, the greater phase stability and fracture toughness of transition metal oxide coatings make them far superior to conventional metallic or organic oxide coatings.	Since powders can become contaminated with the milling media used to grind them, especially when long and repetitive milling cycles are done, it is difficult to generate discrete nanoparticles in the lowest size range.
Biomaterials	They are designed to have some sort of interaction with the body to aid, enhance, or replace natural functions. They aim to heal damaged tissue in the body by tapping into the body’s regenerative capacity through the merging of materials engineering and biological science.	These composites are lighter than conventional ones because high levels of stiffness and strength may be achieved with considerably less high-density materials. The barrier properties of these modified polymers are improved above those of the unmodified counterpart. Their biocompatibility and performance are significantly higher than those of conventional or microstructure materials.	Some of the problems that need to be fixed include the unknown cytotoxicity, the structural integrity, the mechanical characteristics, the corrosion properties, and the long-term stability and service of the components.

## Data Availability

The data presented in this study are available within the article.
